# A Method for Calculating Residual Strength of Crack Arrest Hole on Tungsten-Copper Functionally Graded Materials by Phase-Field Gradient Element Combined with Multi-Fidelity Neural Network

**DOI:** 10.3390/ma18091973

**Published:** 2025-04-26

**Authors:** Bowen Liu, Yisheng Yang, Guishan Wang, Yin Li

**Affiliations:** China Aerodynamics Research and Development Center, Manyang 621000, China; 15b918025@hit.edu.cn (B.L.); ethan_yang@hust.edu.cn (Y.Y.); wangguishan10@nudt.edu.cn (G.W.)

**Keywords:** tungsten-copper functionally graded materials, residual strength assessment, phase-field fracture element, multi-fidelity neural network

## Abstract

This study develops a computational framework for evaluating the residual strength of tungsten-copper functionally graded materials following crack-arrest hole drilling. The proposed methodology features two pivotal innovations: First, a phase-field isoparametric gradient elements is established through representing the gradient effect within the finite element stiffness matrices, incorporating both Amor and Miehe elastic energy decomposition schemes to address tension-compression asymmetry in crack evolution. Second, a multi-fidelity neural network strategy is integrated with the gradient phase-field element to mitigate characteristic length dependency in residual strength predictions. Comparative analyses demonstrate that the gradient finite element achieves smoother field transitions at element interfaces compared to conventional homogeneous elements, as quantified in both stress and damage fields. The Miehe decomposition scheme outperforms the Amor model in capturing complex crack trajectories. Validation against the average strain energy criterion indicates the present approach enhances residual strength prediction accuracy by 39.07% to 44.05%, establishing a robust numerical tool for damage tolerance assessment in graded materials.

## 1. Introduction

The assessment of fracture in notched components is a significant mechanical issue. Notches are primarily sources of stress concentration and resultant structural strength reduction, but individual notches also exhibit reinforcing behavior towards existing cracks [1]. A typical example is the crack arrest hole, which serves as a simple repair solution for U-shaped and V-shaped notches (drilling a circular hole at the tip of the notch to inhibit the continuous expansion of cracks). This method has been widely applied in engineering fields [2,3,4].

Functionally Graded Materials (FGM) are novel composite materials composed of two or more materials, exhibiting continuous gradient variation in composition and structure. Tungsten-copper functional gradient alloy is a typical example of FGMs, featuring a large contrast in melting points and significant differences in structural strength. These unique properties render tungsten-copper alloys extensively utilized in thermal protection and high-voltage electrical contact applications.

Salavati, et al. introduced a notch root area for calculating the average strain energy and employed it as a failure criterion in extensive research on the residual strength evaluation of tungsten-copper alloy notches [5,6,7,8]. Simultaneously, they discovered through experiments that the initial stage of notch fracture in tungsten-copper alloy falls under the category of brittle fracture, providing a physical foundation for the application of the brittle phase field method in this study [8].

In recent years, the phase field method has garnered widespread attention and rapid development in the field of fracture mechanics. Its application range includes but is not limited to fracture [9], fatigue [10], and dynamic [11], and the materials involved include elastoplastic materials, composite materials [12], and even gradient materials [13]. Compared with traditional dispersed crack models, the phase field fracture model can explicitly track crack propagation through phase field variables. Additionally, as it eliminates the need for additional tracking of crack geometry, the phase field fracture model holds unique advantages over the separated crack model in calculating complex crack propagation paths, such as three-dimensional crack propagation and crack branching.

However, from the phase field damage control equation, it can be observed that the characteristic width and fracture energy density impact the crack energy component. When comparing numerous simulation results, it is found that the smaller the characteristic length, the more the damage calculation results exhibit ductile properties, leading to significantly non-conservative strength assessment results. Although the relationship between fracture strength and characteristic width on metals and ceramics has been derived by several scholars [11,14,15,16,17], the relationship is not definite for the complex multi-axial stress state. Meanwhile, for small-sized computational domains, researchers prefer a smaller characteristic width because it allows the calculated crack trajectory to appear more refined. Due to the aforementioned controversy over the value of characteristic width, the phase field method has demonstrated remarkable success in computing crack trajectories, yet its application in the realm of strength assessment appears to be less fruitful.

The primary research emphasis of this paper lies in addressing and optimizing this limitation. In the wake of the rapid advancement of machine learning, neural networks have exhibited immense potential in the data optimization domain [18,19,20,21,22]. Specifically, the multi-fidelity neural network [23], a cost-effective supervised learning model, has attracted the attention of the author due to its capability to achieve data optimization by leveraging a limited quantity of experimental data in conjunction with a substantial volume of simulation data.

The present article focuses on the residual strength assessment of tungsten-copper functionally gradient materials after hole-drilling for crack arrest. The preset paper derives phase-field gradient elements for calculating crack propagation trajectories. Additionally, the influence of different elastic energy decomposition methods and characteristic widths were compared. A method is proposed to reduce the sensitivity of characteristic width on residual strength calculations by integrating phase-field gradient model and multi-fidelity neural network, while simultaneously optimizing the calculation accuracy.

## 2. The Present Method Introduction

### 2.1. Phase Field Gradient Finite Element

Almost all physical control equations originate from the law of conservation of energy, and the principle of minimum potential energy derived from it is the main approach to establishing the weak form of finite elements. Phase field fracture quantifies the fracture energy by introducing a hypothesis of a diffuse crack morphology and integrating this hypothetical crack diffuse area with the fracture energy density. Furthermore, variational methods are utilized to establish both the weak and strong forms of the finite element formulation. The representation is as follows.

(1) $$\Psi^{t}(u,\phi )=\Psi^{e}(u,\phi )+\Psi^{\Gamma }(\phi )$$

where the total energy of the system, $\Psi^{t}(u,\phi )$, is composed of two main components: elastic energy, $\Psi^{e}(u,\phi )$, and fracture energy, $\Psi^{\Gamma }(\phi )$. u represents displacement, and ϕ represents damage degree (with a range of 0–1).

Elastic energy arises from the deformation of the material. Within a certain mechanical domain *Ω*, it can be represented by the integral of the product of strain energy and a damage function.

(2) $$\Psi^{e}(u,\phi )=\int_{\Omega }g(\phi )\psi_{0}(\varepsilon (u))dV$$

where g(ϕ) is the damage function, $\psi_{0}(\varepsilon (u))$ is the strain energy density, which depends on the strain ε in the material, and can be derived from the constitutive equations.

Based on the principles of thermodynamics, the stress-strain relationship can be derived by taking the partial derivative of the strain energy.

(3) $$\sigma =\frac{\partial \psi_{0}(\varepsilon (u))}{\partial \varepsilon }$$

Generally, for linear elastic materials, the strain energy density and the stress-strain relationship can be expressed as follows:

(4) $$\psi_{0}(\varepsilon )=\frac{1}{2}\varepsilon^{T}C\varepsilon$$

In this context, C represents the stiffness matrix expressed in Voigt notation.

The damage function g(ϕ) is a scalar field that depends on the phase field variable ϕ. It accounts for the reduction in stiffness and strength of the material due to damage. The damage function typically takes values between 0 (no damage) and 1 (fully damaged). The function satisfies the following properties:

(5) $$g(0)=1,g(1)=0,g^{\prime }(1)=0$$

In the present paper, the damage degradation function proposed by the AT2 model is selected and written as follows:

(6) $$g(\phi )={\left(1-\phi \right)}^{2}+k$$

where *k* is a parameter chosen to be as small as possible to keep the system of equations well-conditioned. A value of *k* = 1 × 10^−7^ is adopted throughout this work.

**Figure 1:**
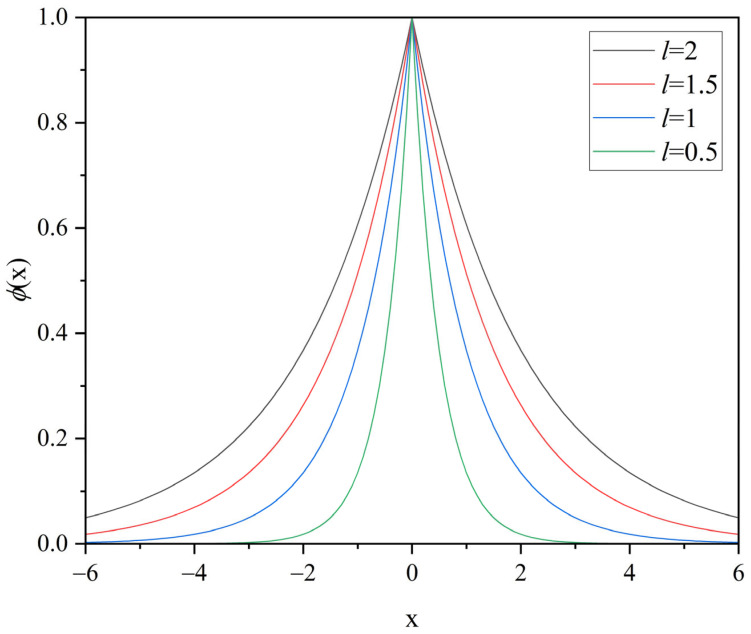
Diffuse representation of a crack at *x* = 0 for various characteristic widths.

For hypothetical diffuse cracks, it is first necessary to choose an appropriate function to describe them. Among a series of candidate functions, the exponential function has been selected.

(7) $$\phi (x)=exp(-\frac{\left|x\right|}{l})$$

where *l* is the length scale parameter that describes the hypothetical diffuse crack width, named phase-field characteristic width. The exponential function happens to satisfy the equation conditions, as shown in Figure 1.

(8) ϕ(x=0)=1,  ϕ(x)→0∶x→±∞

Based on the crack function and employing the principle of minimum potential energy, the problem can be transformed by constructing a variational equation whose solution is the crack function. Hold this idea in mind, the corresponding variational equation can be constructed straightforwardly.

(9) $$\Gamma_{l}(\phi )=\int_{-\infty \;}^{+\infty }\frac{1}{2}[\frac{1}{l}\phi^{2}+l{\left(\nabla \phi \right)}^{2}]dx=\int_{-\infty \;}^{+\infty }\gamma (\phi ,\nabla \phi )dx$$

The integral part of (9) is referred to as the crack area density function $\Gamma_{l}(\phi )$. When multiplied by the critical energy release rate $G_{c}$, which quantifies the energy required to produce a crack of unit size, the fracture energy $\Psi^{\Gamma }(\phi )$ is constituted. The total energy can be rewritten as:

(10) $${\Psi^{t}(u,\phi )=\Psi }^{e}+G_{c}\Gamma_{l}(\phi )=\int_{\Omega }\left\{{g(\phi )\psi }_{0}\left[\varepsilon (u)\right]+\frac{G_{c}}{2}\left[\frac{1}{l}\phi^{2}+l{\left(\nabla \phi \right)}^{2}\right]\right\}dV$$

(11) $$\delta \Psi^{t}(u,\phi )=\int_{\Omega }\left\{\sigma \delta \varepsilon -2(1-\phi )\delta \phi \psi_{0}(\varepsilon )+G_{c}\left[\frac{1}{l}\phi \delta \phi +l\nabla \phi \cdot \nabla \delta \phi \right]\right\}dV$$

The corresponding variational Equation (11) can be derived by applying the variational principle to Equation (10), representing the total energy.

(12) $$\int_{\Omega }\left\{-2(1-\phi )\delta \phi \psi_{0}(\varepsilon )+G_{c}\left[\frac{1}{l}\phi \delta \phi +l\nabla \phi \cdot \nabla \delta \phi \right]\right\}dV=0$$

(13) $$\int_{\Omega }\left\{\sigma \delta \varepsilon -b\cdot \delta u\right\}dV+\int_{\partial \Omega }h\cdot \delta udA=0$$

To discretize phase-field partial differential equations by finite element method, the weak form of damage (12) and displacement (13) should be obtained, respectively.

Where **b** and **h** are the body and surface forces acting on the computation domain, respectively.

In order to establish the stiffness matrix of the finite element, displacement, strain and damage variables can be discretized by interpolation functions.

(14) $$u=\sum_{i=1}^{m}N_{i}^{u}u_{i},\;\delta u=\sum_{i=1}^{m}N_{i}^{u}{\delta u}_{i},\;\delta \varepsilon =\sum_{i=1}^{m}B_{i}^{u}{\delta u}_{i}\;\;\;\;\;\;\;\;\;\phi =\sum_{i=1}^{m}N_{i}^{\phi }\phi_{i},\;\delta \phi =\sum_{i=1}^{m}N_{i}^{\phi }{\delta \phi }_{i},\;\nabla \delta \phi =\sum_{i=1}^{m}B_{i}^{\phi }{\delta \phi }_{i}$$

where *m* represents the number of element nodes, and the corner mark *i* represents the corresponding node number. N is the shape function. $B^{u}$ is the strain-displacement matrices, and $B^{\phi }$ is the gradient matrix of interpolation function.

In order to reduce the numerical step on the element interface and realize the accurate calculation of the stress field and damage field of gradient material, this paper is inspired by the gradient isoparametric element proposed by Paulino [24], introducing the shape function into the expression of gradient material property and establishing the phase field gradient isoparametric element. In detail, to characterize the gradient effect in the stiffness matrix of the finite element, the elastic model *E*, Poisson’s ratio υ, and fracture energy density *G_c_* are discretized by following the discrete method of strain and damage through interpolation functions, which are expressed as follows:

(15) $$E=\sum_{i=1}^{m}N_{i}E_{i},\;\upsilon =\sum_{i=1}^{m}N_{i}\upsilon_{i},\;G_{c}=\sum_{i=1}^{m}N_{i}{G_{c}}_{i}$$

The tungsten-copper functionally graded material studied in this paper belongs to the case of linear elastic plane strain, and the elastic stiffness matrix C  can be expressed as:

(16) $$C=\frac{\sum_{i=1}^{m}N_{i}E_{i}}{\left(1+\sum_{i=1}^{m}N_{i}\upsilon_{i})(1-2\sum_{i=1}^{m}N_{i}\upsilon_{i}\right)}\left[\begin{array}{ccc} 1-\sum_{i=1}^{m}N_{i}\upsilon_{i} & \sum_{i=1}^{m}N_{i}\upsilon_{i} & 0\\ \sum_{i=1}^{m}N_{i}\upsilon_{i} & 1-\sum_{i=1}^{m}N_{i}\upsilon_{i} & 0\\ 0 & 0 & \frac{1-2\sum_{i=1}^{m}N_{i}\upsilon_{i}}{2} \end{array}\right]$$

Similarly, the residual with respect to the evolution of the phase field damage and displacement can be expressed respectively:

(17) $$r_{i}^{\phi }=\int_{\Omega }\left\{-2(1-\phi )N_{i}^{\phi }\psi_{0}(\varepsilon )+G_{c}\left[\frac{1}{l}N_{i}^{\phi }\phi +l{\left(B_{i}^{\phi }\right)}^{T}\nabla \phi \right]\right\}dV$$

(18) $$r_{i}^{u}=\int_{\Omega }[{\left(1-\phi \right)}^{2}+k]{\left(B_{i}^{u}\right)}^{T}C\varepsilon dV-\int_{\Omega }{\left(N_{i}^{u}\right)}^{T}bdV-\int_{\partial \Omega }{\left(N_{i}^{u}\right)}^{T}hdA$$

The Newton–Raphson method is employed to obtain the solutions for $r_{i}^{\phi }=0$ and $r_{i}^{u}=0$, and thus the finite element damage–displacement stiffness matrix is represented as (19).

(19) $$\left\{\begin{array}{c} u\\ \phi \end{array}\right\}={\left\{\begin{array}{c} u\\ \phi \end{array}\right\}}_{t}-\left[\begin{array}{cc} \frac{\partial r^{u}}{\partial u} & \frac{\partial r^{u}}{\partial \phi }\\ \frac{\partial r^{\phi }}{\partial u} & \frac{\partial r^{\phi }}{\partial \phi } \end{array}\right]\left\{\begin{array}{c} r^{u}\\ r^{\phi } \end{array}\right\}$$

### 2.2. Elastic Energy Decomposition Scheme

To distinguish the differential contributions of tension and compression to damage, a series of elastic energy decomposition methods have been proposed. Among them, the most classic ones are the Amor volumetric separation model [25] and the Miehe spectral decomposition model [26]. The separation method decomposes the elastic energy portion to focus the damage on tensile deformation.

(20) $$\psi_{0}=g(\phi )\psi^{+}+\psi^{-}$$

where $\psi^{+}$ and $\psi^{-}\;$ represent the positive and negative parts of the strain energy, respectively.

The specific form of the Amor model is as follows:

(21) $$\psi^{\pm }=\frac{1}{2}K{\langle tr(\varepsilon )\rangle }_{\pm }^{2}+H(\pm 1)\mu (\varepsilon^{\prime }∶\varepsilon^{\prime })$$

while the Miehe spectral decomposition model is given by:

(22) $$\psi^{\pm }=\frac{1}{2}\lambda {\langle tr(\varepsilon )\rangle }_{\pm }^{2}+\mu \varepsilon^{\pm }∶\varepsilon^{\pm }$$

where *K* denotes the bulk modulus, λ and µ are the Lame parameters, a±=12(a±a), *H* is Heaviside function, $\varepsilon^{\prime }$ is deviator strain, and $\varepsilon^{+}$ and $\varepsilon^{-}$ are tensile and compressive principal strains, respectively.

The positive and negative components of the stress and stiffness matrices are the first and second partial derivatives of the corresponding elastic corresponding variables, respectively.

(23) $$\sigma^{\pm }=\frac{\partial \psi^{\pm }}{\partial \varepsilon }{,\;C}^{\pm }=\frac{\partial \sigma^{\pm }}{\partial \varepsilon }$$

Concretely, for the Amor model, taking tr(ε)≥0 as an example, the positive component of stress is expressed as follows:

(24) $${\sigma^{+}(\varepsilon )}_{ij}=\frac{\partial \psi^{+}(\varepsilon )}{\partial \varepsilon_{ij}}=\frac{K}{2}2tr(\varepsilon )\frac{\partial \left[tr(\varepsilon )\right]}{\partial \varepsilon_{ij}}+2\mu \varepsilon^{\prime }∶\frac{\partial \varepsilon^{\prime }}{\partial \varepsilon_{ij}}$$

The stress–strain relationship can be represented by a constant matrix, from which the stiffness matrix $C^{+}$ can be obtained:

(25) $$\left[\begin{array}{c} \sigma_{1}^{+}\\ \sigma_{2}^{+}\\ \begin{array}{c} \sigma_{3}^{+}\\ \sigma_{4}^{+}\\ \begin{array}{c} \sigma_{5}^{+}\\ \sigma_{6}^{+} \end{array} \end{array} \end{array}\right]=\left\{\underset{C^{+}}{\underbrace{K\left[\begin{array}{cccccc} 1 & 1 & 1 & & & \\ 1 & 1 & 1 & & & \\ 1 & 1 & 1 & & & \\ & & & 0 & & \\ & & & & 0 & \\ & & & & & 0 \end{array}\right]+2\mu \left[\begin{array}{cccccc} \frac{2}{3} & -\frac{1}{3} & -\frac{1}{3} & & & \\ -\frac{1}{3} & \frac{2}{3} & -\frac{1}{3} & & & \\ -\frac{1}{3} & -\frac{1}{3} & \frac{2}{3} & & & \\ & & & 1 & & \\ & & & & 1 & \\ & & & & & 1 \end{array}\right]}}\right\}\left[\begin{array}{c} \varepsilon_{1}\\ \varepsilon_{2}\\ \begin{array}{c} \varepsilon_{3}\\ \varepsilon_{4}\\ \begin{array}{c} \varepsilon_{5}\\ \varepsilon_{6} \end{array} \end{array} \end{array}\right]$$

The stiffness matrix expression can be obtained when tr(ε)<0 in the identical manner.

As to Miehe model, the strain tensor can be expressed as the combination of principal component and characteristic component.

(26) $$\varepsilon =\sum_{a=1}^{3}\varepsilon_{a}{\hat{N}}^{a}⊗{\hat{N}}^{a},\;\varepsilon^{+}=\sum_{a=1}^{3}\varepsilon_{a}^{+}{\hat{N}}_{a}⊗{\hat{N}}_{a}$$

where subscript *a* denotes the principal strain index, and $\hat{N}$ represents the corresponding eigenvectors.

Taking the positive part of stress as an example, the expression can be deduced as:

(27) $$\sigma^{+}(\varepsilon )=\frac{\partial \psi^{+}(\varepsilon )}{\partial \varepsilon }=\lambda \;\langle tr(\varepsilon )\rangle \frac{\partial \langle tr(\varepsilon )\rangle }{\partial \varepsilon }+\mu \varepsilon^{+}∶\frac{\partial \varepsilon^{+}}{\partial \varepsilon }=\lambda \;\langle tr(\varepsilon )\rangle H(tr(\varepsilon ))1+2\mu \varepsilon^{+}$$

The key point of calculating stiffness matrix is the derivative of normal strain tensor and total strain tensor, which can be expressed as a fourth-order tensor [27]:

(28) $$P^{+}=\frac{\partial \varepsilon^{+}}{\partial \varepsilon }=\sum_{a=1}^{3}\frac{\partial \lambda_{a}^{+}}{\partial \lambda_{a}}{\hat{N}}^{a}⊗{\hat{N}}^{a}⊗{\hat{N}}^{a}⊗{\hat{N}}^{a}+\sum_{\begin{array}{c} a=1,b=1\\ a\neq b \end{array}}^{3}\frac{\lambda_{b}^{+}-\lambda_{a}^{+}}{2(\lambda_{b}-\lambda_{a})}{\hat{N}}^{a}⊗{\hat{N}}^{b}⊗({\hat{N}}^{a}⊗{\hat{N}}^{b}+{\hat{N}}^{b}⊗{\hat{N}}^{a})$$

Then the expression of stiffness matrix positive part $C_{ijkl}^{+}\;$ can be derived as:

(29) $$C_{ijkl}^{+}=\frac{\partial \sigma^{+}}{\partial \varepsilon }=\lambda H\left[tr(\varepsilon )\right]\delta_{ij}\delta_{kl}+2\mu P_{ijkl}^{+}$$

### 2.3. Decoupling Between Displacement and Damage

By employing a historical, monotonically increasing variable $H_{t}$ to track the maximum value of the positive part of elastic energy, decoupling between displacement and damage can be achieved, which indicates that:

(30) $$H_{t}=\left\{\begin{array}{c} \psi^{+}(\varepsilon )\;\;\;\;\;if\;\psi^{+}(\varepsilon )>H_{t}\\ H_{t}\;\;\;\;\;\;otherwise\;\; \end{array}\right.$$

The decoupled residual with respect to the evolution of the phase field damage and displacement can be overwritten respectively by introducing $H_{t}$.

(31) $$r_{i}^{u}=\int_{\Omega }[{\left(1-\phi \right)}^{2}+k]{\left(B_{i}^{u}\right)}^{T}C^{+}\varepsilon dV+{\left(B_{i}^{u}\right)}^{T}C^{-}\varepsilon dV-\int_{\Omega }{\left(N_{i}^{u}\right)}^{T}bdV-\int_{\partial \Omega }{\left(N_{i}^{u}\right)}^{T}hdA$$

(32) $$r_{i}^{\phi }=\int_{\Omega }\left\{-2(1-\phi )N_{i}^{\phi }H_{t}+G_{c}\left[\frac{1}{l}N_{i}^{\phi }\phi +l{\left(B_{i}^{\phi }\right)}^{T}\nabla \phi \right]\right\}dV$$

Thus, only the diagonal non-zero component in Newton-Raphson method is retained in the element stiffness matrix [18], as expressed:

(33) $${KK}_{i}^{u}=\int_{\Omega }\left[{\left(1-\phi \right)}^{2}+k\right]{\left(B_{i}^{u}\right)}^{T}C^{+}\varepsilon dV+{\left(B_{i}^{u}\right)}^{T}C^{-}\varepsilon dV-\int_{\Omega }{\left(N_{i}^{u}\right)}^{T}bdV-\int_{\partial \Omega }{\left(N_{i}^{u}\right)}^{T}hdA$$

(34) $$K_{ij}^{\phi \phi }=\frac{\partial r_{i}^{\phi }}{\partial \phi_{j}}=\int_{\Omega }\left\{\left[2H_{t}+\frac{G_{c}}{l}\right]N_{i}^{\phi }N_{j}^{\phi }+G_{c}l{\left(B_{i}^{\phi }\right)}^{T}B_{j}^{\phi }\right\}dV$$

**Figure 2:**
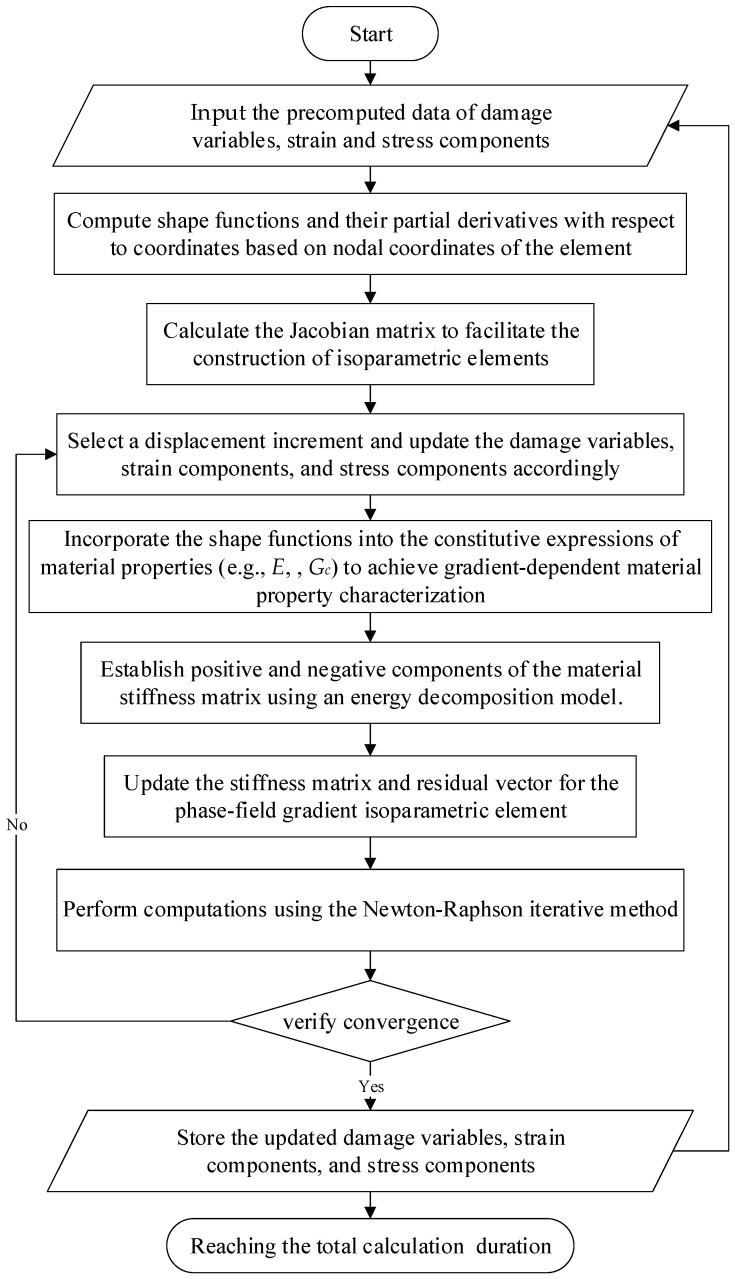
Numerical implementation procedure of phase-field gradient isoparametric element.

Specifically, to integrate the two energy decomposition models into the phase-field framework, the graded material properties are first computed based on the material parameters (e.g., *E*, υ, *G_c_*) through Equations (15) and (16). These properties are subsequently applied to derive parameters (the bulk modulus *K*, and the Lame parameters λ, µ) in the energy decomposition models (Equations (21) and (22)), thereby enabling the determination of the positive and negative parts of **C**. Finally, the phase-field-displacement coupled finite element calculations are implemented through Equations (31)–(34) to complete the numerical formulation. At this point, the gradient properties are expressed in the element stiffness matrix expression. The numerical calculations of phase field gradient isoparametric elements are implemented through the UEL subroutine of the commercial software ABAQUS 2016. The Numerical implementation procedure of phase-field gradient isoparametric element is shown in Figure 2.

### 2.4. Phase Field Gradient Finite Element Combined with Multi-Fidelity Neural Network

Material strength testing is a type of consumptive mechanical experiment. Since the inception of interest in strength research, the focus has been on how to utilize limited experimental data to achieve high-accuracy strength predictions.

**Figure 3:**
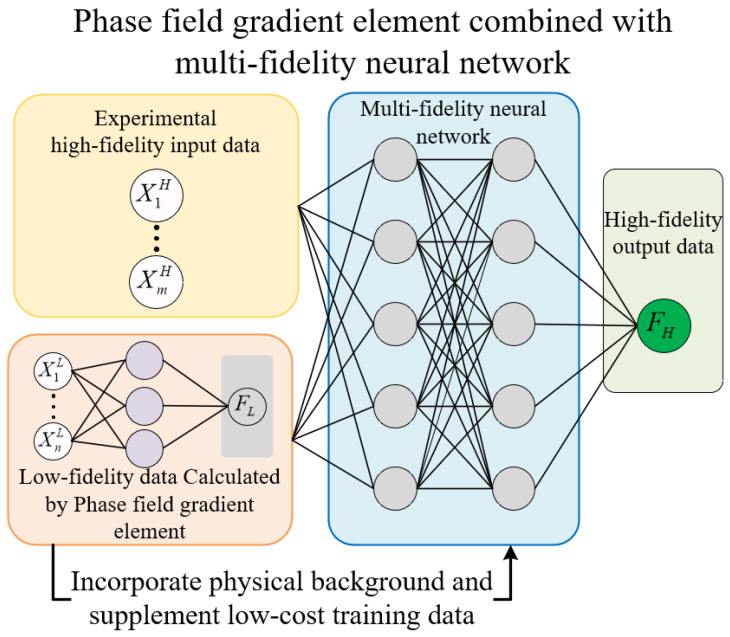
Phase-field gradient element combined with multi-fidelity neural network.

Multi-fidelity neural networks can effectively leverage “low-fidelity data” obtained through low-cost methods (such as numerical simulations and semi-empirical models) to investigate its correlation with “high-fidelity” experimental data, thereby optimizing the final output accuracy. The residual strength prediction method using this feature combined with the phase-field gradient finite element is shown in Figure 3.

The model consists of two parts. The first part is a “low-fidelity” data training model based on a fully connected neural network. In this part, the geometric characteristics of the notch are used as input data, and the residual strength calculated by the phase-field gradient finite element serves as the “low-fidelity” output data. Through training, the “low-fidelity” input–output relationship can be obtained, allowing for the acquisition of a large amount of physically meaningful “low-fidelity” output data. The second part is a multi-fidelity neural network that takes experimental data as “high-fidelity” data and combines it with the “low-fidelity” output data as input. By training the neural network to find the relationship between high- fidelity and low-fidelity data, it achieves a fusion of physical damage mechanisms with machine learning. Ultimately achieving the goal of residual intensity forecasting. The damage function is the sum of the mean square error (*MSE*) of high and low fidelity data (35), and the L2 regularization method is utilized to modify the loss function to reduce training noise and overfitting.

(35) $$\mathrm{MSE}={\mathrm{MSE}}_{\mathrm{NL}}+{\mathrm{MSE}}_{\mathrm{NH}}+\lambda \sum w^{2}\;\;\;\;{\mathrm{MSE}}_{\mathrm{NL}}=\frac{1}{n}\sum {\left(N_{L}-N_{L}^{*}\right)}^{2}\;\;\;\;\;\;\;\;\;\;\;\;\;\;\;\;{\mathrm{MSE}}_{\mathrm{NH}}=\frac{1}{n}\sum {\left(N_{H}-N_{H}^{*}\right)}^{2}$$

where *MSE_NL_* and *MSE_NH_* denotes the mean squared error for the low-fidelity and high-fidelity data, respectively. The expression $\lambda \sum w^{2}$ represents the L2 regularization term, in which λ signifies the weights of the neural network and *w* denotes the regularization coefficient. *n* and *m* are the numbers of low-fidelity and high-fidelity training data samples, respectively. *N_L_* and *N_H_* refer to the low-fidelity and high-fidelity training data sets, while N_L_^*^ and N_H_^*^ represent the low-fidelity and high-fidelity prediction results, respectively.

## 3. Model Validation and Results Discussion

**Figure 4:**
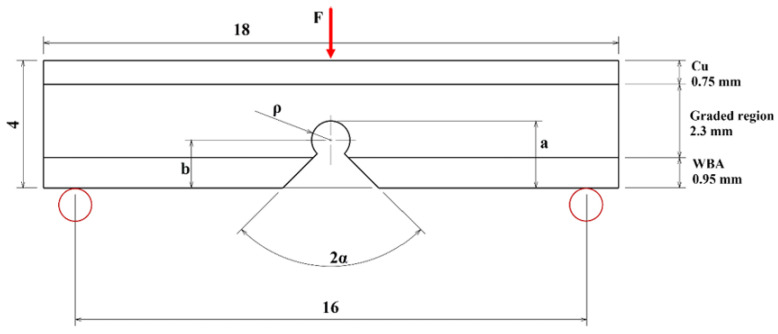
Geometric dimensions, material distribution and loading positions of tungsten-copper functionally graded structure [5].

**Figure 5:**
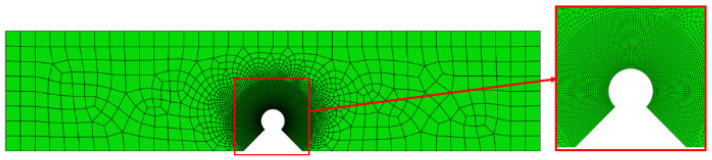
Finite element model with ρ = 0.3 mm and α = 45°.

The validation data in this paper are sourced from literature [5], which documents a tungsten-copper functionally graded alloy fabricated using the powder metallurgy method, and nine different hole-drilled crack arrest configurations were tested. To verify the residual strength of the crack arrest with a hole, the literature adopts a three-point bending test as the evaluation method, which induces MODE I fracture. The material distribution and loading configuration are illustrated in the accompanying Figure 4, and the finite element model is shown in Figure 5.

In the finite element model, the experimental displacement loading was simulated by applying a vertically downward displacement boundary condition of 0.2 mm while constraining the horizontal displacement to zero. Additionally, the vertical degrees of freedom at the support contact locations on the specimen’s lower surface were constrained to replicate the experimental boundary conditions. A mapped meshing technique was employed with an element dimension of 0.01 mm in the notch root. The total simulation duration was set to 1, with a time increment size of 0.0001. Both mesh density and time increment parameters were determined through convergence verification adopting a two-stage refinement approach. For example, in the notch configuration with a radius of 0.3 mm and an opening angle of 60°, the variation in phase-field damage at the notch root remained below 1% when the mesh size was coarsened from 0.02 mm to 0.01 mm at identical simulation time step. Similarly, reducing the time increment from 0.0002 to 0.0001 resulted in a fracture load variation from 86.0 MPa to 85.7 MPa, corresponding to a relative change of less than 0.35%. These results confirm that the selected mesh density and time increment parameters satisfy convergence requirements for numerical reliability.

As is suggested in [5], the power law function was employed to describe mechanical properties of the graded region, and thus the elasticity modulus E, Poisson ratio v, ultimate tensile stress $\sigma_{ut}$, and fracture toughness $K_{Ic}$, as expressed by Equation (36) and Table 1.

For the plane strain mentioned in this article, based on the theory of fracture mechanics, the failure stress is attained *K_Ic_* with Griffith’s critical energy release rate: $G_{c}=\frac{\left(1-\upsilon^{2}\right)K_{IC}^{2}}{E}$ as suggested in ref. [13].

(36) $$E\left(Z\right)=\left[E_{Cu}-E_{WBA}\right]{\left(\frac{2Z+h}{2h}\right)}^{n}+E_{WBA}\;v\left(Z\right)=\left[v_{Cu}-v_{WBA}\right]{\left(\frac{2Z+h}{2h}\right)}^{n}+v_{WBA}\;\sigma_{ut}\left(Z\right)=\left[{\sigma_{ut}}_{Cu}-{\sigma_{ut}}_{WBA}\right]{\left(\frac{2Z+h}{2h}\right)}^{n1}+{\sigma_{ut}}_{WBA}\;K_{Ic}\left(Z\right)=\left[{K_{Ic}}_{Cu}-{K_{Ic}}_{WBA}\right]{\left(\frac{2Z+h}{2h}\right)}^{n2}+{K_{Ic}}_{WBA}$$

where *Z* is the thickness coordinate of the graded region (−*h*/2 < *Z* < *h*/2) and *h* is the thickness of the graded region (*h* = 2.3 mm).

### 3.1. Calculation of Stress and Damage Field

This study employs phase-field isoparametric gradient elements to conduct finite element analysis on tungsten-copper gradient notched specimens. The computational results are systematically compared with those derived from the conventional homogeneous element method, thereby validating the superior computational accuracy of the proposed isoparametric gradient element formulation.

In light of the conclusion from the literature [24] on stress isoparametric elements—“Higher-order graded elements (e.g., quadratic and higher) are superior to conventional homogeneous elements based on the same shape functions” and “One should be careful when using graded elements with linear shape functions (e.g., Q4) as it may lose accuracy in certain situations such as uniform loading parallel to the material gradient direction”—the present study employs an 8-node phase-field isoparametric planar element (Q8 Graded) for comparative computations, in contrast to conventional homogeneous elements. The homogeneous element formulation adopted in this study is consistent with that described in [24]. The homogeneous element properties are defined by the material parameters at the element centroid, which are implemented through a user-defined material subroutine (UMAT) in ABAQUS.

In addition to the baseline Mode I loading case (uniform loading parallel to the material gradient direction), this investigation additionally incorporates uniform displacement loading perpendicular to the gradient direction as supplementary validation scenarios. The material properties of the comparative homogeneous phase-field elements (Q8 Homog) are determined by the material parameter values corresponding to the geometric centroids.

**Figure 6:**
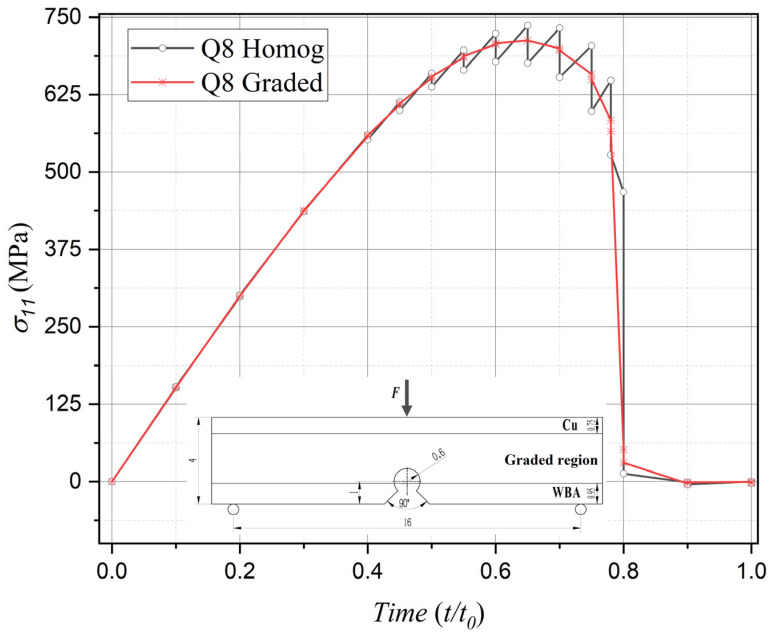
Comparison of stress component evolution between phase-field graded isoparametric elements and homogeneous elements under Mode I loading.

**Figure 7:**
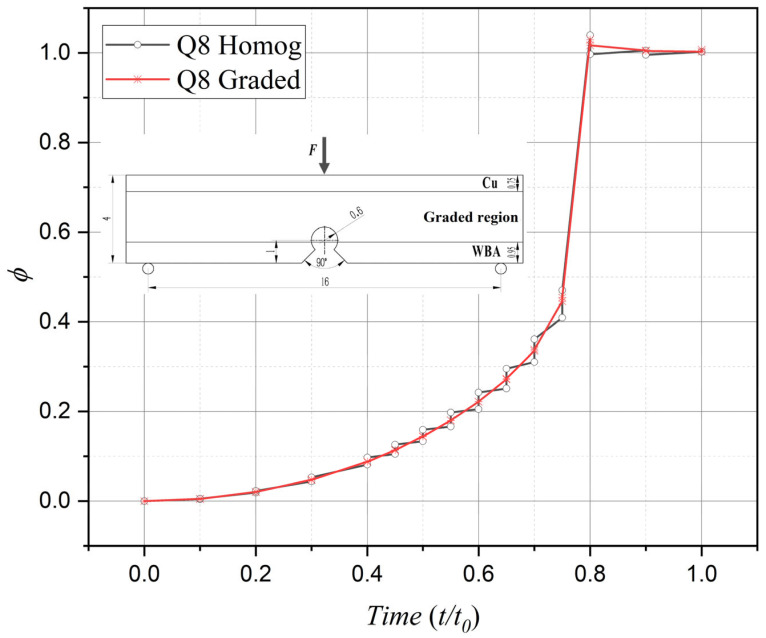
Comparison of damage evolution between phase-field graded isoparametric elements and homogeneous elements under Mode I loading.

**Figure 8:**
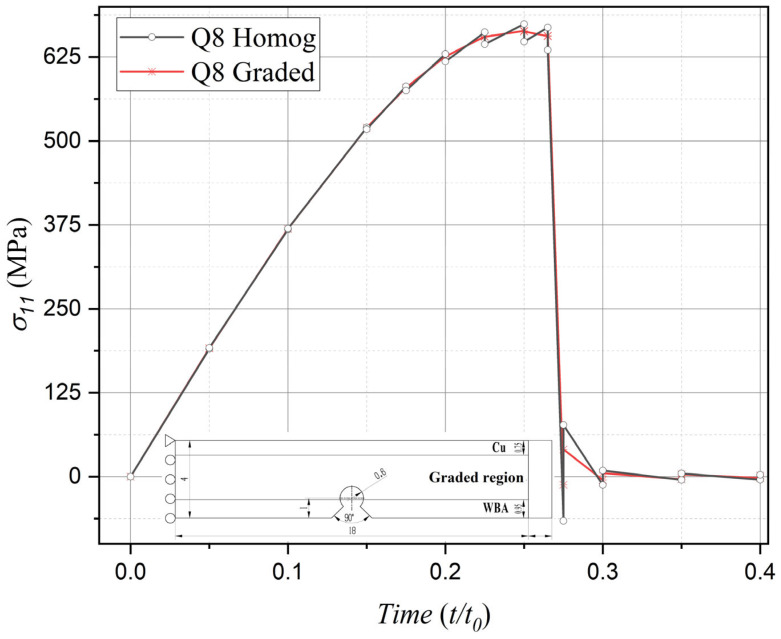
Comparison of stress component evolution between phase-field graded isoparametric elements and homogeneous elements under horizontal uniform displacement loading.

**Figure 9:**
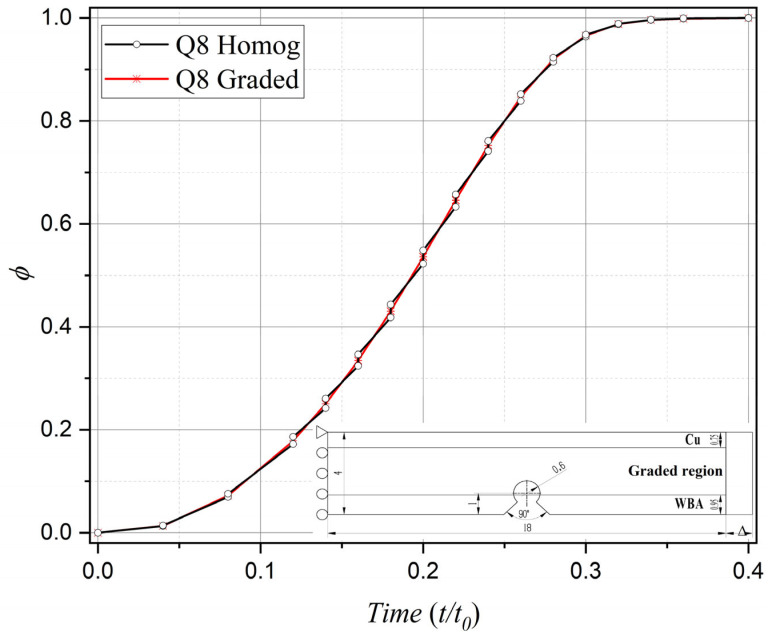
Comparison of damage evolution between phase-field graded isoparametric elements and homogeneous elements under horizontal uniform displacement loading.

The comparative analysis specifically targets the interface nodes between two elements located along the vertical central axis, positioned closest to the notch root along the material gradient direction. The comparative results are illustrated in Figure 6, Figure 7, Figure 8 and Figure 9, where the horizontal axis represents the normalized computational time, expressed as *t*/*t*_0_, where *t* denotes the current simulation time instant and *t*_0_ = 1 indicates the total simulation duration. Specifically, regarding the horizontal stress component *σ*_11_ (the primary driving force for Mode I fracture) under Mode I loading, both element formulations exhibit stress evolution profiles characterized by an initial ascent followed by subsequent descent, which aligns with the physical progression of fracture damage. However, within the 0.4–0.8 temporal range, the homogeneous element solution demonstrates pronounced stress oscillations associated with stepwise discontinuities across element interfaces—a phenomenon conflicting with physical reality. Conversely, the graded element formulation substantially mitigates such oscillations, demonstrating superior numerical stability and enhanced solution accuracy.

Similarly, in terms of phase-field damage evolution, computational results from both element types progressively converge toward the critical value of 1 with increasing load cycles, consistent with actual fracture processes. Nevertheless, comparative analysis reveals that the graded element formulation achieves significantly improved computational fidelity over its homogeneous counterpart, particularly during the critical 0.4–0.8 temporal progression phase.

Moreover, under displacement loading conditions perpendicular to the material gradient distribution, computational results obtained from homogeneous elements manifest pronounced oscillations across element interfaces during the 0.15–0.27 temporal window—a phenomenon observable in both stress field distributions and damage evolution patterns. In marked contrast, the proposed phase-field graded isoparametric element formulation demonstrates consistent computational robustness, accurately resolving interfacial mechanical components throughout the entire simulation duration. This comparative evidence further validates the enhanced reliability of the present graded element methodology in handling complex loading configurations.

### 3.2. Calculation of Crack Propagation Path

Unlike from tensile strength loading, which involves a simple uniaxial tensile stress state, three-point bending loads generate both compressive and tensile stresses on the cross-sections. When addressing such fracture problems using the phase-field method, it is necessary to distinguish the different contributions of tensile and compressive stresses to damage evolution, as done in elastic energy decomposition models. Therefore, this paper first focuses on two scenarios with different combinations of hole radii and opening angles. Calculations are performed using the Amor model and the Miehe model. A comparative analysis is then conducted to assess the effectiveness of these different models in calculating the path of Mode I cracks.

**Figure 10:**
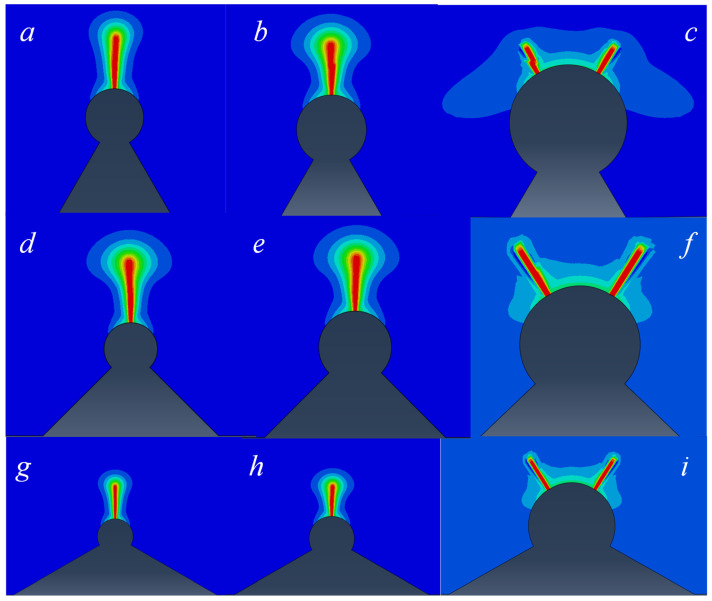
Crack propagation path calculated by phase field gradient element combined with Amor model. ((**a**) ρ = 0.3 mm 2α = 60°, (**b**) ρ = 0.4 mm 2α = 60°, (**c**) ρ = 0.6 mm 2α = 60°, (**d**) ρ = 0.3 mm 2α = 90°, (**e**) ρ = 0.4 mm 2α = 90°, (**f**) ρ = 0.6 mm 2α= 90°, (**g**) ρ = 0.3 mm 2α = 120°, (**h**) ρ = 0.4 mm 2α = 120°, (**i**) ρ = 0.6 mm 2α = 120°).

For the studied tungsten-copper gradient alloy, the crack path calculated adopting Amor model is shown in the Figure 10 it can be observed that regardless of the change in the opening angle, the crack propagation for small-radius circular notches (radii = 0.3 mm and 0.4 mm) exhibits a Mode I crack propagation path, i.e., it extends along the extension line of the root of the notch. However, in the case of a larger circular notch (with a notch radius of 60 mm), instead of following a Mode I crack propagation path, the calculation results show a crack path that forms an angle similar to 45° with the median line.

**Figure 11:**
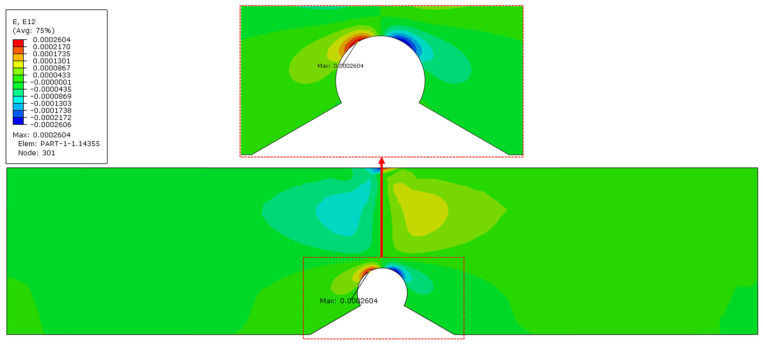
Shear strain distribution in the undamaged state.

The reasons for this abnormal result are as follows. From the strain distribution in the undamaged state (Figure 11), it can be observed that the shear strain in this region is relatively high, which is the main component of the deviatoric strain. However, the equations of Amor model (21) show that, although the model does distinguish between positive and negative volumetric strain components, it neglects the distinction between deviatoric strain components. Coupled with the distribution characteristics of the tungsten-copper gradient alloy, which has a higher copper alloy content towards the top, resulting in a higher energy requirement for cracking, and this leads to the emergence of 45-degree evolution behavior.

On the contrary, the Miehe model employs a representation in terms of components in the principal strain space, which exhibits a notable capability in distinguishing between positive and negative strains, encompassing both volumetric strain regions and deviatoric strain components.

**Figure 12:**
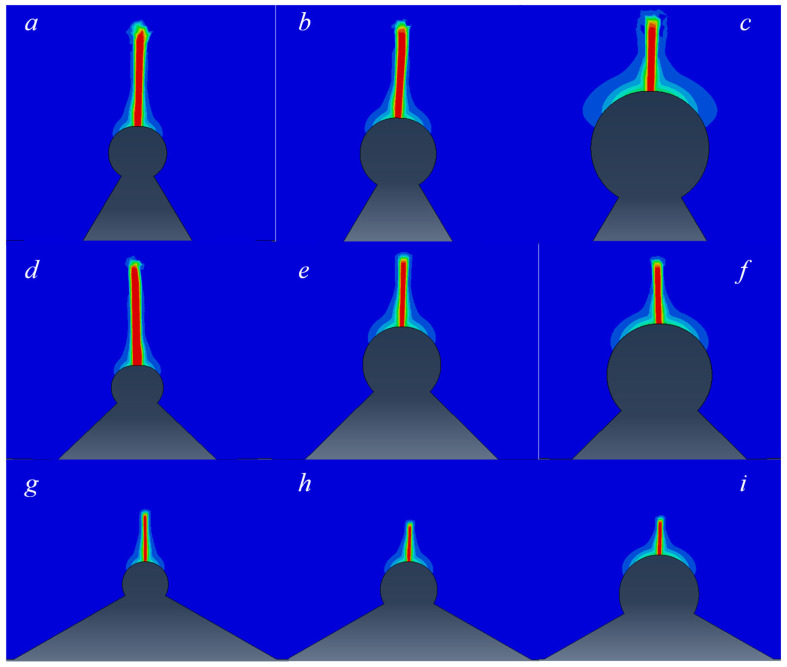
Crack propagation path calculated by phase field gradient element combined with Miehe model. ((**a**) ρ = 0.3 mm 2α = 60°, (**b**) ρ = 0.4 mm 2α = 60°, (**c**) ρ = 0.6 mm 2α = 60°, (**d**) ρ = 0.3 mm 2α = 90°, (**e**) ρ = 0.4 mm 2α = 90°, (**f**) ρ = 0.6 mm 2α = 90°, (**g**) ρ = 0.3 mm 2α = 120°, (**h**) ρ = 0.4 mm 2α = 120°, (**i**) ρ = 0.6 mm 2α = 120°).

The crack path calculation results of the Miehe model are illustrated in the Figure 12. The figure illustrates that the Miehe model accurately captures the Mode I crack propagation for all notch radii and opening angles, which is characterized by crack trajectory collinear with the applied force direction. This alignment with experimental reality underscores the validity and reliability of Miehe model in simulating and predicting crack behavior in the tungsten-copper gradient functional material.

### 3.3. Sensitivity of Residual Strength to Characteristic Width

**Figure 13:**
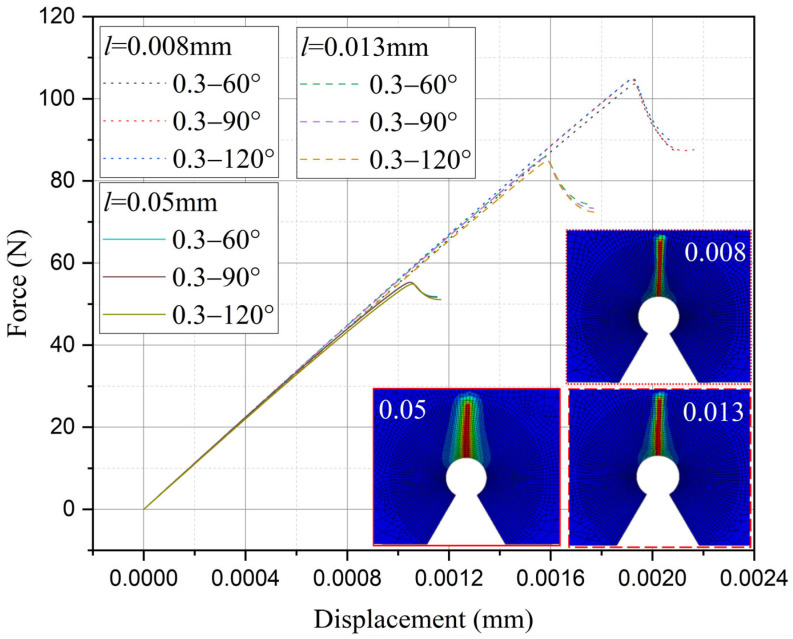
Load history calculated with different characteristic widths (ρ = 0.3 mm, 2α from 60° to 120°).

**Figure 14:**
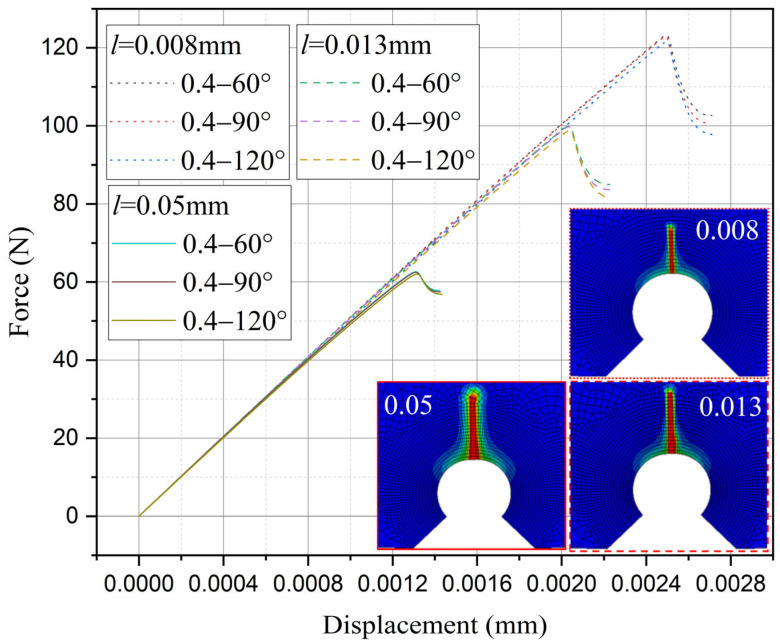
Load history calculated with different characteristic widths (ρ = 0.4 mm, 2α from 60° to 120°).

**Figure 15:**
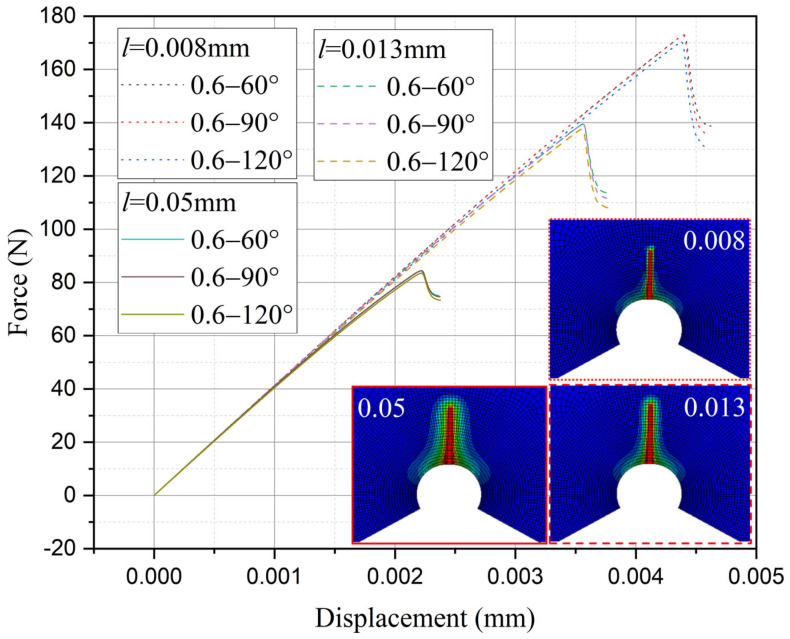
Load history calculated with different characteristic widths (ρ = 0.6 mm, 2α from 60° to 120°).

**Figure 16:**
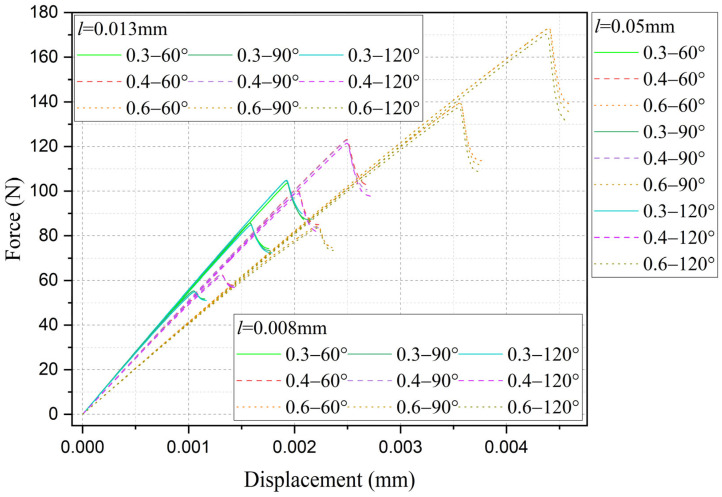
Load history calculated with different characteristic widths (notch radius ρ from 0.3 mm to 0.6 mm, opening angle 2α from 60° to 120°).

Furthermore, the influence of characteristic width on the residual strength of the notch tungsten-copper gradient functional materials is discussed. Three characteristic widths of 0.008 mm, 0.013 mm, and 0.05 mm are adopted to calculate the 9 notch shapes mentioned above, and the results are shown in Figure 13, Figure 14, Figure 15 and Figure 16.

It can be observed that the loading force trajectory trends of the same notch geometry are consistent, and the residual strength (maximum load force value) gradually increases with the decrease of characteristic width. The maximum increase is close to 400 MPa, corresponding to a notch radius of 0.3 mm, when the characteristic width is reduced from 0.05 mm to 0.008 mm. And the crack path indeed appears thinner.

As described in previous studies [27] and the strong form equation of phase field damage, *l* and *G_C_* are important parameters that affect damage evolution. Unfortunately, *l* is a hypothetical diffuse crack width rather than the actual crack width, which gives it great flexibility in selecting values. On the other hand, although *G_C_* is a material constant, its quantitative measurement is very difficult. Even when derived from traditional fracture mechanics parameters such as *K_IC_*, it is constrained by the geometry of the notch and the stress state.

Despite the aforementioned limitations with the phase field method in strength assessment, for each characteristic width, the trend in the calculated results of residual strength for different notches is consistent. Furthermore, this trend aligns with the distribution trend observed in experimental data. This consistency suggests that machine learning algorithms, which excel at identifying and exploiting patterns in data, could be effectively applied to refine and improve the accuracy of the phase field method strength predictions in subsequent section.

### 3.4. Optimization Results of Multi-Fidelity Neural Networks

In this section, a multi-fidelity neural network is employed to integrate and train two datasets with similar trends, aiming to establish a correlation between phase-field numerical results and experimental outcomes, ultimately achieving the refinement of residual strength assessment.

For high-fidelity data, two sets of experimental data are adopted: one with a notch radius ρ = 0.3 mm and angle 2α = 60°, and the other with ρ = 0.6 mm and 2α = 120°. The notch radius and crack depth (characterized by the opening angle) serve as input data. The low-fidelity neural network consists of four hidden layers, each with 10 neurons, while the high-fidelity neural network has two hidden layers, each also with 10 neurons. The input layer dimensions for high fidelity and low fidelity are both 2 (notch radius and opening angle), and the output layer dimension is 1 (residual intensity). The dimensions of the input and output layers of a multi fidelity neural network are both 1. L2 regularization term is adopted with λ equal to 0.01, accompanying Glorot uniform as weight initialization scheme. The hyperbolic tangent function is selected as the activation function the mathematical form as f(x)=tanh(x). Employing the Adam optimizer as the model optimization, with an iterative step length set at 0.001, and the entire computation process encompasses 80,000 steps. The subsequent training error history can determine that the setting of the learning rate can achieve effective convergence in training. The specific mechanism of information flow between the networks is described as follows: First, the low-fidelity model is trained using the input-output pairs from low-fidelity data to obtain low-fidelity outputs. Subsequently, both the high-fidelity outputs and the obtained low-fidelity outputs are used as inputs to the multi-fidelity neural network, while the high-fidelity output data serve as the target outputs for training the multi-fidelity neural network. Ultimately, this process yields the final multi-fidelity outputs.

To verify that the method can eliminate the sensitivity of residual strength to characteristic width, training and learning are conducted based on the data with different values of *l*, and the optimization results are compared thereafter.

**Figure 17:**
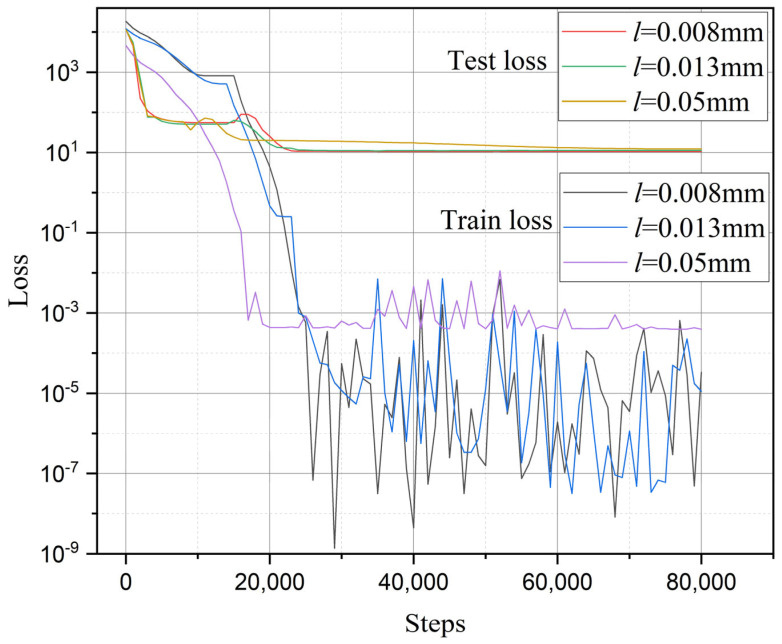
Training and validation error histories of different characteristic widths.

The training and validation error histories are illustrated in the Figure 17. It can be observed that the validation errors corresponding to different characteristic widths converge to nearly the same value (within a range of 10.8 to 12.4), indicating that the multi-fidelity neural network indeed reduces the dependence of phase-field strength results on the characteristic width *l*.

**Figure 18:**
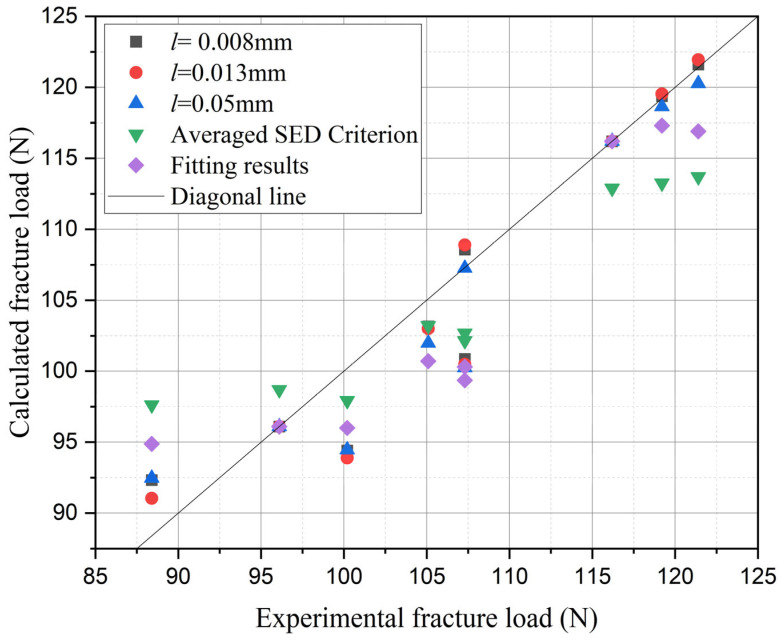
Comparison of notch residual strength calculated by the average strain energy criterion, fitting-based approach and the present method with 3 characteristic widths.

The training results are illustrated in the Figure 18, demonstrating that even with only two sets of experimental data, the phase-field numerical calculation results have been significantly corrected. The maximum error range between the corrected results and the experimental data is only 6.5%. The inherent architecture of multi-fidelity neural networks ensures that training accuracy scales positively with the quantity of high-fidelity data samples (e.g., experimental measurements). Consequently, the predictive accuracy of the proposed model can be further enhanced through the systematic integration of additional experimental datasets into the training protocol.

Furthermore, a comparison between the results calculated by the present method and those derived from the average strain energy criterion is illustrated in the Figure 18. It can be observed that the proposed method exhibits a higher precision. To quantify the optimization effect, the overall relative error was introduced for the data not selected for supervised training. The definition of relative error is as follows:

(37) $$Relative\;Error=\sum \frac{\left|Calculated\;fracture\;load-Experimental\;fracture\;load\right|}{Experimental\;fracture\;load}\cdot 100\%$$

where the calculated fracture load and experimental fracture load correspond to the numerical values shown in Figure 18.

The calculation results, as depicted in the Figure 18, indicate that the average strain energy criterion yields an overall relative error of 34.94. In contrast, the model proposed in this paper exhibits an overall relative error ranging from 19.55 to 21.29. This represents a minimum improvement of approximately 39.07% to 44.05% (calculated as [(34.94 − 21.29)/34.94]100% and [(34.94 − 19.55)/34.94]100%, respectively) compared to the average strain energy criterion. This significant reduction in error underscores the effectiveness and superiority of the method introduced in this paper.

**Figure 19:**
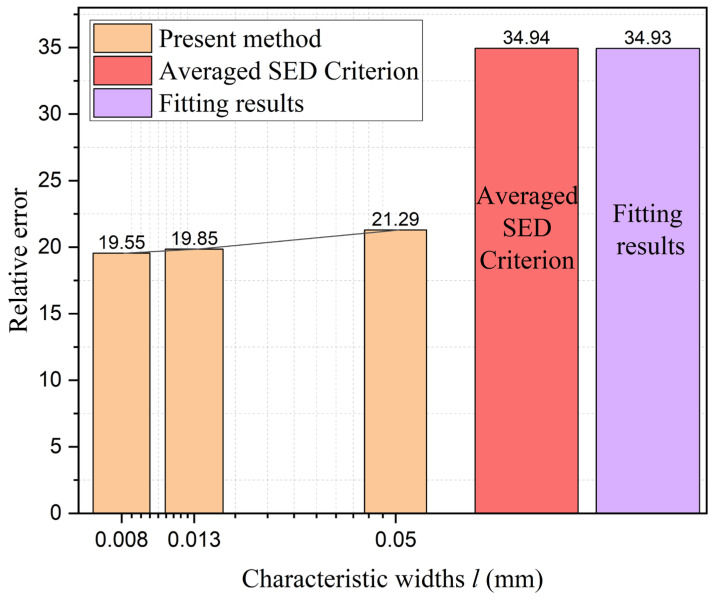
Comparison of relative error of the average strain energy criterion, fitting-based approach and the present method with 3 characteristic widths.

In addition, to demonstrate the advantages of the multi-fidelity neural network model adopted in this study, a comparison was made between the present model and a fitting-based approach. To ensure fairness in the comparison, the fitting approach was also applied to the identical two sets of high-fidelity data used in the neural network model (notch radius 0.3 mm with an opening angle of 60°, and notch radius 0.6 mm with an opening angle of 120°). Then the phase-field characteristic width was determined as 0.016 mm for first data and 0.028 mm for second data through iterative trial-and-error calculations. As revealed by Figure 16 and further elaborated in the subsequent section, the notch radius was identified as the predominant factor governing residual strength. Leveraging the two calculated datasets, a linear interpolation scheme was implemented to establish the relationship between notch radius and characteristic width. This relationship was then extrapolated to compute the residual strength for nine distinct notch configurations. The comparison between the calculated results and experimental data is presented in Figure 17 and Figure 18, labeled as fitting results. As demonstrated by the relative error (Equation (37)) values shown in Figure 19, the computational accuracy of the fitting method is comparable to that of the average strain energy criterion. Moreover, the multi-fidelity neural network model demonstrates a significant improvement in accuracy over the fitting method, approximately 39.05% to 44.03% (calculated as [(34.93−21.29)/34.93]100% and [(34.93−19.55)/34.93]100%, respectively). This enhanced performance stems from the ability of neural network to account for not only the influence of notch radius but also the opening angle and inherent characteristic width, further validating its superiority in capturing multiple parameter dependencies.

In addition, to validate the generalizability of the proposed model across broader datasets, the average strain energy density from the literature [5] was adopted as high-fidelity training data, while the computational results from the phase-field gradient isoparametric elements served as low-fidelity data. The neural network architecture and hyperparameters remained identical to the aforementioned configurations. As the strain energy density method revealed negligible influence of opening angles on residual strength, only notch radius and depth were considered as input parameters. The comparative results are tabulated in Table 2, where the average strain energy density data marked with an asterisk (*) denote the 10 high-fidelity training data points. Whole 64 notch configurations were evaluated adopting the phase-field gradient elements (PFG) to generate low-fidelity data. The result of training the multi fidelity neural network (MFNN) coupled with the phase field gradient element is represented as PFG with MFNN.

**Figure 20:**
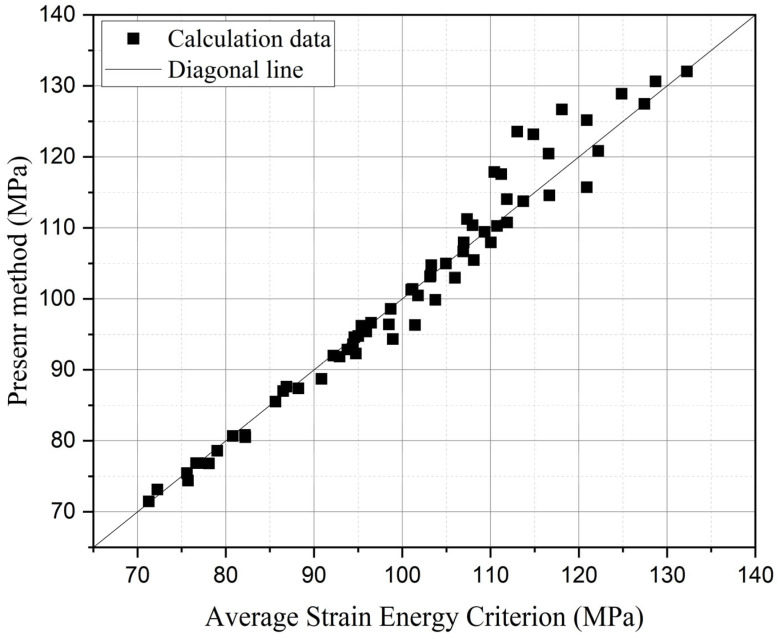
Comparison of the present method and average strain energy criterion.

**Figure 21:**
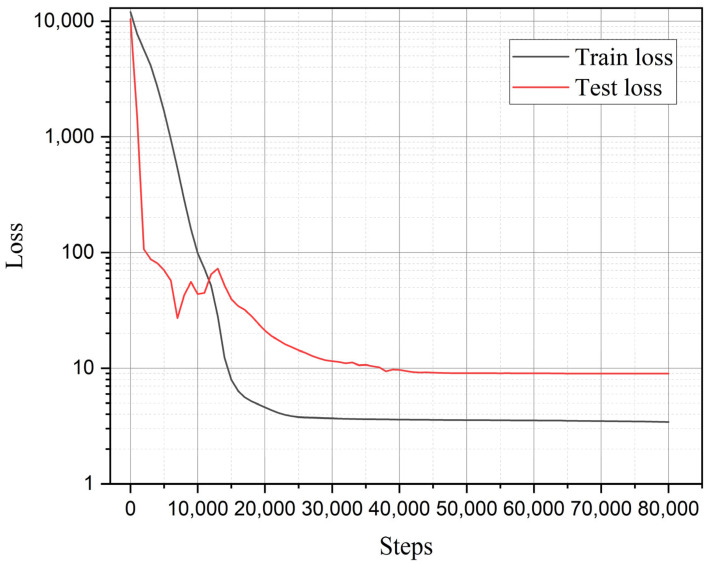
Training and validation error histories of the present method on comparison data.

A comparative analysis of residual strength predictions between the present model and the average strain energy criterion is presented in Figure 20, while the training error evolution of the multi-fidelity neural network is illustrated in Figure 21. As shown in Figure 20, the residual strength results from both methods cluster near the diagonal, indicating that the MFNN optimization framework, which leverages a limited high-fidelity data alongside extensive phase field simulations, achieves predictions consistent with high-fidelity benchmarks across a broad parameter space. The MFNN training history in Figure 21 reveals final converged training and validation errors of 3.43 and 8.98, respectively. Comparative analysis with Figure 16 demonstrates a systematic reduction in training error as the volume of high-fidelity data increases, a trend that aligns with the inherent characteristics of multi-fidelity neural networks.

### 3.5. The Influence of the Notch Size on the Fracture Strength

**Figure 22:**
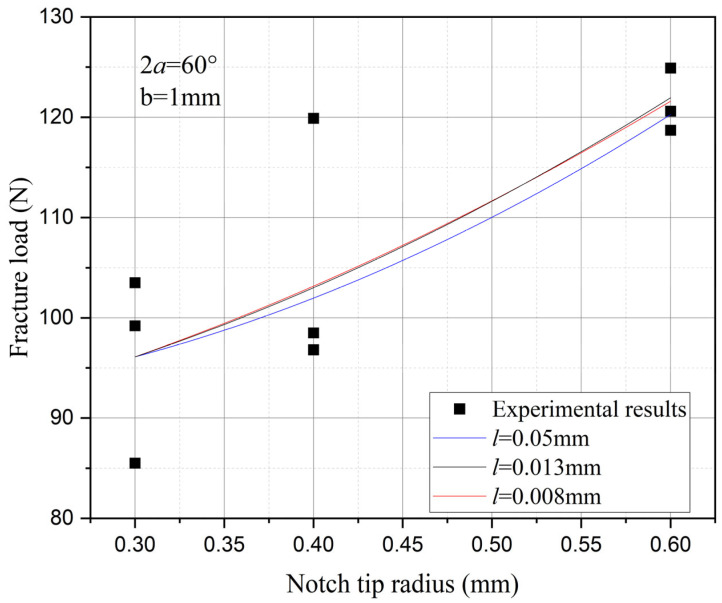
The influence of the notch tip radius on the fracture strength with the opening angle (2α = 60°) and fixed depth.

**Figure 23:**
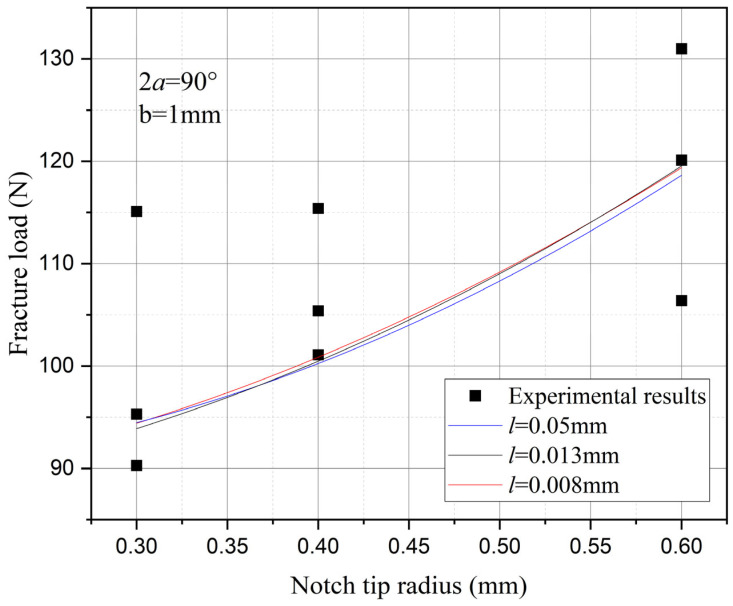
The influence of the notch tip radius on the fracture strength with the opening angle (2α = 90°) and fixed depth.

**Figure 24:**
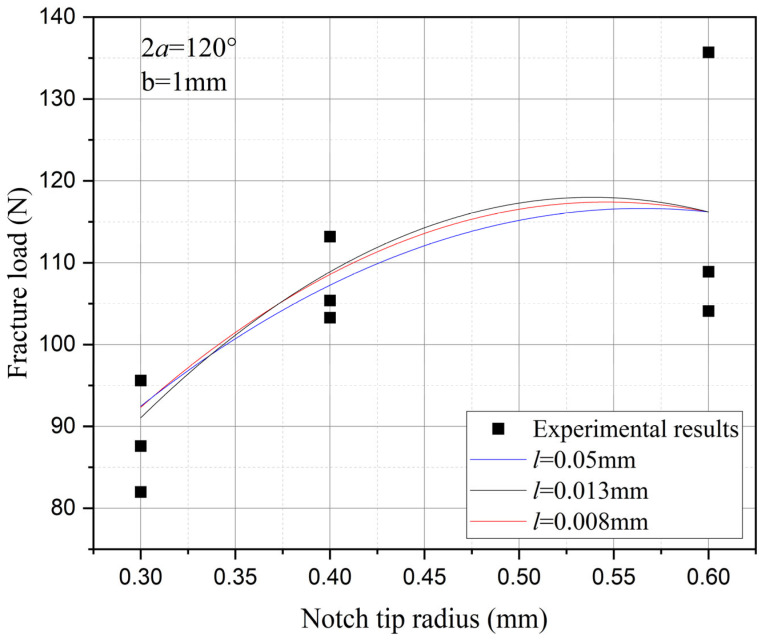
The influence of the notch tip radius on the fracture strength with the opening angle (2α = 120°) and fixed depth.

**Figure 25:**
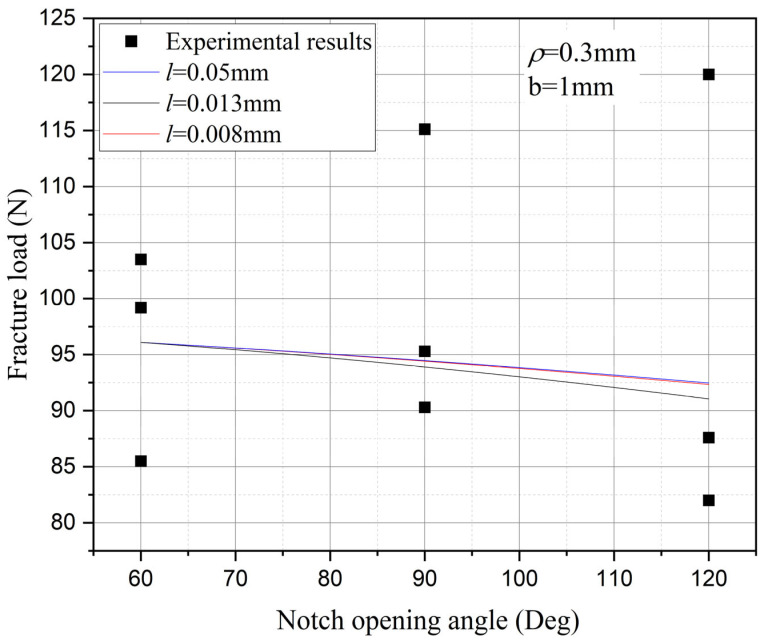
The influence of the notch opening angle on the fracture strength with the notch tip radius (ρ = 0.3 mm) and fixed depth.

**Figure 26:**
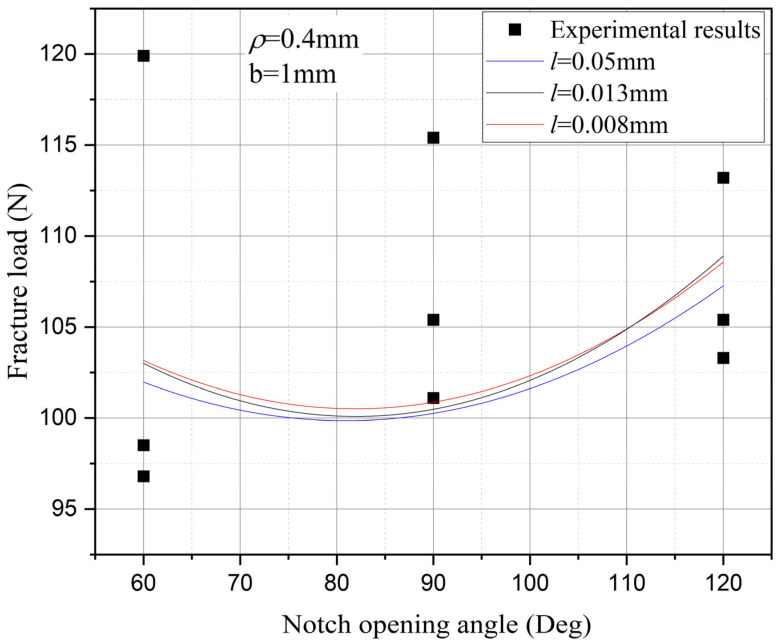
The influence of the notch opening angle on the fracture strength with the notch tip radius (ρ = 0.4 mm) and fixed depth.

**Figure 27:**
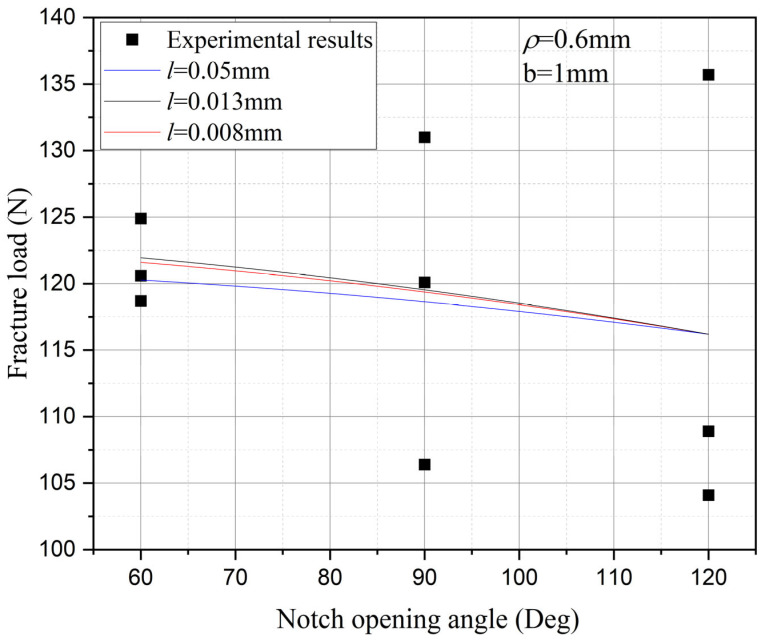
The influence of the notch opening angle on the fracture strength with the notch tip radius (ρ = 0.6 mm) and depth fixed.

Based on the computational results of the present method, the influence of the notch size on the fracture strength of tungsten-copper functional gradient materials is analyzed. First, fix the opening angle and crack depth, and compare the influence of notch radius on fracture strength. From Figure 22, Figure 23 and Figure 24, it can be observed that the fracture strength significantly increases with the increase of the notch radius within the range of 0.3–0.6 mm. This is completely opposite to the fracture properties of traditional homogeneous materials. Second, with the notch radius and depth fixed, the influence of the notch angle on the notch fracture strength is compared. Figure 25, Figure 26 and Figure 27 show that the fracture strength is not particularly sensitive to changes in the notch angle. This phenomenon is also consistent with the findings presented in Figure 13, Figure 14, Figure 15 and Figure 16. The strength calculation model proposed in this article can provide data support for the content (such as: design optimization, scalability, or structural health monitoring systems), which will be carried out in subsequent research.

## 4. Conclusions

The present study addresses the residual strength evaluation of tungsten-copper functionally graded materials (FGMs) with crack-arresting holes. The main conclusions have been summarized as follows:

1. A phase-field graded isoparametric element is formulated by embedding gradient characterization into the stiffness matrix constitutive framework. The proposed graded elements are superior to conventional homogeneous elements based on the same shape functions in the calculation of stress and damage fields.
2. Compared with the Amor model, the Miehe model combined with graded element can accurately predict crack propagation paths, types, and residual strength distribution trends.
3. The characteristic width is the predominant factor influencing the strength evaluation. The proposed method can reduce the sensitivity of the residual strength to the characteristic width by combining the phase field graded finite element with the multi-fidelity neural networks.
4. For the studied tungsten-copper functional gradient material, within a certain range, a larger notch unexpectedly results in higher residual strength. The methodology presented in this paper can accurately characterize this abnormal phenomenon.

## Figures and Tables

**Table 1:** Mechanical properties of boundary layers.

Boundary Regions	Elasticity Modulus *E* (GPa)	Poisson’s Ratio *ν*	Ultimate Tensile $\sigma_{ut}$ (MPa)	Fracture Toughness *K_Ic_* (MPa.m ^0.5^)	*n*	*n*_1_	*n*_2_
Cu	112.7	0.34	188.9	88.5	0.9646	1.312	2.78
WBA	289.5	0.29	447.3	4.52			

**Table 2:** Calculation results of PFG, SED and PFG with MFNN.

Model No.	ρ (mm)	a (mm)	PFG (MPa)	SED (MPa)	PFG with MFNN (MPa)
1 *	0.05	1	70.88	76.64	76.87
2	0.1	1	79.21	85.63	85.54
3	0.15	1	86.90	95.36	96.22
4	0.2	1	93.94	103.14	103.14
5	0.25	1	100.34	110.73	110.28
6	0.3	1	106.10	116.66	114.58
7	0.35	1	111.21	122.19	120.85
8	0.4	1	115.68	127.43	127.48
9 *	0.45	1	119.50	132.24	132.04
10 *	0.05	1.05	69.15	75.60	75.47
11 *	0.1	1.1	76.25	80.80	80.68
12	0.05	1.15	66.04	72.25	73.15
13	0.1	1.15	75.24	79.04	78.60
14	0.15	1.15	83.37	86.87	87.64
15 *	0.2	1.15	90.52	94.56	94.62
16	0.25	1.15	96.79	101.17	101.40
17	0.3	1.15	102.30	106.95	107.96
18	0.35	1.15	107.14	111.84	114.07
19	0.4	1.15	111.40	116.58	120.47
20	0.45	1.15	115.20	120.90	125.19
21	0.5	1.15	118.63	124.84	128.90
22	0.55	1.15	121.80	128.70	130.63
23	0.2	1.2	90.87	92.18	92.01
24	0.25	1.25	100.02	95.92	95.43
25	0.05	1.3	70.11	71.28	71.47
26	0.1	1.3	77.99	75.72	74.39
27	0.15	1.3	85.28	82.21	80.52
28	0.2	1.3	91.98	88.22	87.40
29	0.25	1.3	98.08	93.78	92.87
30 *	0.3	1.3	103.59	98.69	98.61
31	0.35	1.3	108.50	103.28	104.76
32	0.4	1.3	112.82	107.32	111.25
33	0.45	1.3	116.54	111.20	117.59
34	0.5	1.3	119.67	114.84	123.18
35	0.55	1.3	122.20	118.08	126.69
36	0.6	1.3	124.14	120.90	115.74
37	0.35	1.35	110.40	101.00	101.29
38 *	0.4	1.4	117.50	103.21	103.31
39	0.05	1.45	74.70	76.87	76.86
40	0.1	1.45	82.87	78.08	76.83
41	0.15	1.45	90.53	82.17	80.84
42	0.2	1.45	97.67	86.50	87.02
43	0.25	1.45	104.30	90.84	88.73
44	0.3	1.45	110.41	94.74	92.30
45	0.35	1.45	116.01	98.47	96.42
46	0.4	1.45	121.10	101.77	100.49
47 *	0.45	1.45	125.67	104.93	104.99
48	0.5	1.45	129.73	107.96	110.38
49	0.55	1.45	133.27	110.43	117.88
50	0.6	1.45	136.30	113.02	123.56
51	0.5	1.5	133.50	106.90	106.70
52	0.55	1.55	151.02	109.30	109.45
53	0.05	1.60	70.12	95.01	94.79
54 *	0.1	1.6	84.03	92.89	91.83
55	0.15	1.6	95.98	94.41	93.62
56	0.2	1.6	106.20	96.45	96.65
57	0.25	1.6	114.93	98.91	94.35
58	0.3	1.6	122.42	101.43	96.33
59	0.35	1.6	128.90	103.74	99.87
60	0.4	1.6	134.61	105.96	102.98
61	0.45	1.6	139.80	108.09	105.48
62	0.5	1.6	144.70	110.03	107.97
63	0.55	1.6	149.55	111.87	110.77
64 *	0.6	1.6	154.60	113.70	113.76

Symbol * denote the 10 high-fidelity training data points.

## Data Availability

The original contributions presented in this study are included in the article. Further inquiries can be directed to the corresponding author.
